# The Management of Esophageal Perforation: A Systematic Review

**DOI:** 10.7759/cureus.63651

**Published:** 2024-07-02

**Authors:** Tariq M Shaqran, Rawan Engineer, Esra M Abdalla, Abdulrahman A Alamoudi, Reham Almahdi, Ahmed Aldhahri, Afyaa M Alghamdi, Bashair M Abufarea, Ruyuf F Almutairi, Ali A Al-Suliman

**Affiliations:** 1 Family Medicine, King Salman Armed Forces Hospital, Tabuk, SAU; 2 Surgery, Salmaniya Medical Complex, Manama, BHR; 3 Family Medicine, Michigan State University, East Lansing, USA; 4 College of Medicine, Faculty of Medicine, King Abdulaziz University, Jeddah, SAU; 5 College of Medicine, Al Baha University, Al Baha, SAU; 6 Medicine and Surgery, Ibn Sina National College for Medical Studies, Jeddah, SAU; 7 Medicine and Surgery, Al Baha University, Al Baha, SAU; 8 Medicine and Surgery, Faculty of Medicine, King Abdulaziz University, Jeddah, SAU; 9 College of Medicine, University of Tabuk, Tabuk, SAU; 10 College of Medicine, Vision Colleges, Riyadh, SAU

**Keywords:** perforation, esophageal, boerhaave syndrome, rupture, management

## Abstract

Esophageal perforation, a rare and serious condition, has seen a reduction in mortality from 30% to 15% over the last three decades due to advancements such as gastrointestinal stents, minimally invasive surgeries, and improved interventional radiology techniques. This review analyzes management strategies for esophageal perforation based on 14 English-language articles published from 2009 to 2024, primarily utilizing surveys and national database analyses. The management of esophageal perforation is complex, with challenges in diagnosis and treatment strategy. Despite surgery being the traditional treatment, the role of less invasive methods is growing. Effective management of esophageal perforation involves advanced imaging for diagnosis, hemodynamic stabilization, and a multidisciplinary approach to treatment, including surgical and non-surgical interventions. The evidence for different treatment outcomes remains limited, highlighting the need for comprehensive care involving thoracic surgery, interventional radiology, gastroenterology, and critical care in an intensive care unit setting.

## Introduction and background

Esophageal perforation represents a critical health emergency, characterized by mortality rates as high as 50% [1]. Despite significant strides in diagnostic and treatment methodologies, this condition continues to pose a considerable challenge for healthcare teams. The esophagus, a fibromuscular tube approximately 25 centimeters in length that bridges the pharynx and the stomach, is susceptible to perforations. The most prevalent type of perforation occurs within the intrathoracic region (54%), followed by perforations in the cervical esophagus (27%), and intra-abdominal perforations (19%) [2,3]. In the United States, the incidence of esophageal perforations is approximately three cases per 100,000 individuals annually, with these instances distributed as 25% cervical, 55% intrathoracic, and 20% abdominal [4,5].

Iatrogenic esophageal perforations are primarily induced by medical instruments during diagnostic or therapeutic procedures. Diagnostic endoscopy presents the lowest risk, whereas therapeutic interventions, including pneumatic dilation, hemostasis, stent placement, removal of foreign bodies, cancer palliation, and endoscopic ablation methods, notably increase perforation risk [6]. Such perforations typically occur in the hypopharynx and the distal esophagus. While rare, invasive surgical techniques like fundoplication and esophageal myotomy can also lead to iatrogenic perforations [7].

Spontaneous esophageal ruptures, another category of perforation, typically affect the esophagus's posterolateral wall, resulting from a sudden increase in intra-esophageal pressure against a drop in intrathoracic pressure. Although less common, traumatic esophageal injuries represent a critical, potentially lethal form of esophageal perforation. Perforations due to foreign body impaction are rare, frequently occurring in the distal third of the esophagus. Such impactions can lead to pressure necrosis, resulting in ischemia and necrosis of the wall, eventually causing perforation [4,5].

The esophagus, notable for its absence of a serosal layer, is particularly vulnerable to ruptures and perforations. Such incidents can lead to chemical mediastinitis, marked by inflammation and mediastinal necrosis. Following a full-thickness tear, a polymicrobial bacterial translocation and invasion can ensue within hours, posing a significant risk of sepsis and potentially fatal outcomes without swift intervention. Pleural effusion, manifesting as either sympathetic or exudative, is a possible consequence of esophageal perforation, the occurrence of which hinges on the pleura's integrity. Delay in diagnosis or insufficient medical intervention can escalate to severe complications [8].

Perforations in the cervical region typically manifest as neck pain, dysphagia, odynophagia, or dysphonia. In contrast, perforations in the thoracic area can lead to symptoms such as retrosternal chest pain, retching, and vomiting, particularly noted in individuals with Boerhaave syndrome. During examination, mediastinal crackling may be detected through auscultation, alongside signs of pleural effusion. Abdominal esophageal perforations are likely to cause epigastric pain, possibly accompanied by nausea or vomiting. Full-thickness tears, especially when resulting in peritoneal contamination, may escalate to acute peritonitis. Advanced conditions such as late-stage mediastinitis and shock can be indicated by fever, rapid heart rate (tachycardia), rapid breathing (tachypnea), low blood pressure (hypotension), and bluish skin coloration (cyanosis), all of which are considered negative prognostic factors. The time elapsed from the occurrence of the perforation to the commencement of treatment significantly affects the clinical outcomes and manifestations [9,10].

Plain radiographs can identify air leakage from a perforated esophagus, presenting as subcutaneous emphysema in cases of cervical esophageal rupture, pneumomediastinum and mediastinal widening for thoracic perforations, and free air beneath the diaphragm for abdominal perforations. Contrast esophagography is pivotal for confirming esophageal perforation by revealing contrast leakage and dye extravasation. Although barium contrast studies offer greater accuracy and specificity, water-soluble contrast studies are favored to minimize the risk of barium-induced chemical mediastinitis. For suspected esophageal perforations, a computed tomography (CT) scan of the chest and abdomen is recommended to detect intrathoracic or intra-abdominal fluid collections, necessitating percutaneous or surgical intervention. CT imaging may also show peri-esophageal fluid collections, esophageal wall thickening, and pneumomediastinum [11-13].

The differential diagnosis for esophageal perforation is broad and includes conditions such as acute aortic dissection, acute coronary syndrome, pericarditis, aspiration pneumonitis, various forms of pneumonia (including bacterial pneumonia and abscess pneumonia), pancreatitis, empyema, Mallory-Weiss tear, myocardial infarction, peptic ulcer disease, and pulmonary embolism [8]. The aim of this systematic review is to explore the current management strategies for esophageal perforation, ensuring an accurate diagnosis and effective treatment plan that is applied.

## Review

Methods

This review adhered to the guidelines of the Preferred Reporting Items for Systematic Reviews and Meta-Analyses (PRISMA).

Search Strategy

**Figure 1:**
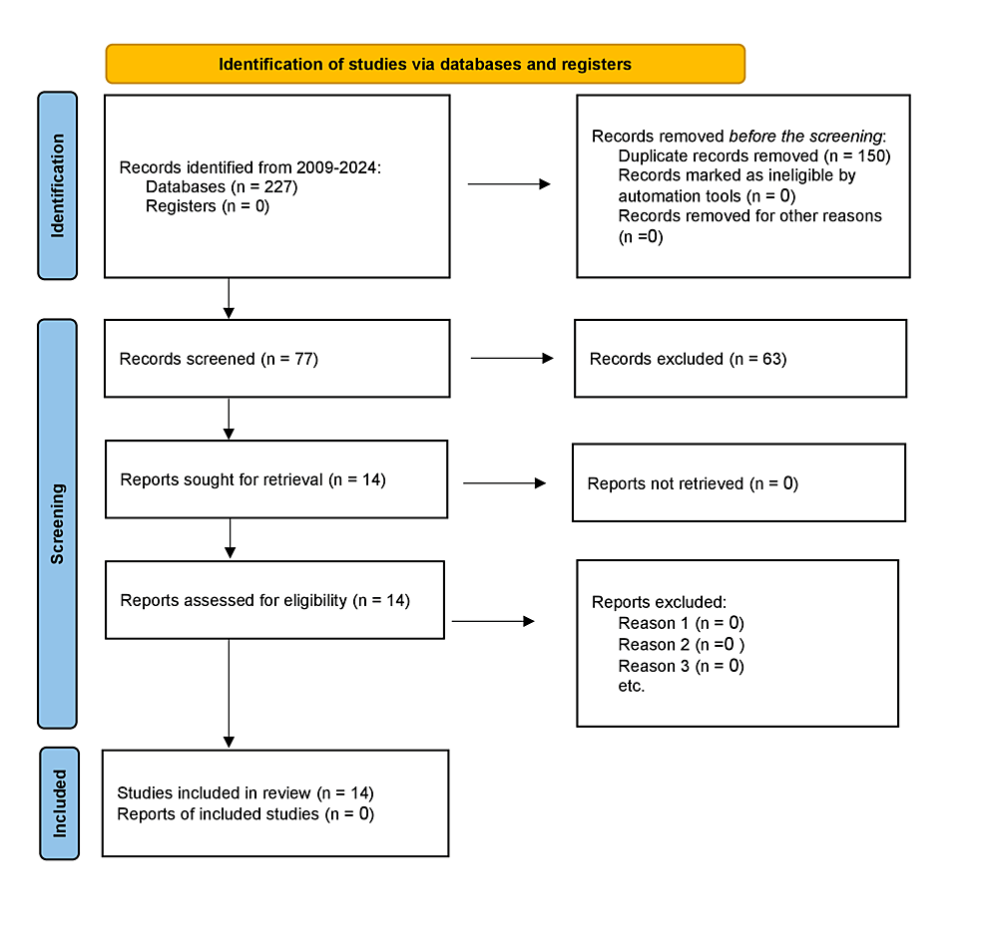
PRISMA study flow diagram PRISMA: Preferred Reporting Items for Systematic Reviews and Meta-Analyses

Keywords and MeSH (Medical Subject Headings) terms were utilized to search PubMed/Medline and Embase databases from 2009 to January 2024, focusing on "esophageal perforation management." Further studies were identified by examining the references of selected articles. Ultimately, 14 articles were deemed relevant for inclusion in this review following the PRISMA guidelines for article selection and screening (Figure 1).

Inclusion and Exclusion Criteria

Included were English-language research articles and reviews addressing esophageal perforation management. The studies required (1) detailed reports on diagnosing esophageal perforation; (2) data integrity and reproducibility; and (3) focus on management approaches.

Exclusion criteria encompassed (1) data that was duplicate, incomplete, erroneous, or not applicable; (2) editorials, conference summaries, and non-research literature; (3) outdated or regionally redundant comprehensive data; (4) studies with unclear sample sources; and (5) non-English publications. Studies conducted before 2009 were also excluded.

Primary Outcome

The principal objective was to assess the management strategies for esophageal perforation.

Data Extraction

An initial search yielded 227 studies, with 77 papers considered after title and abstract screening. A thorough review of the full texts led to the selection of 14 papers for detailed analysis (Table 1).

**Table 1:** A summary of studies, including authors, publication years, causes of esophageal perforation, and management strategies, was compiled.

S. no.	Authors and years	Causes of esophageal perforation	Possible way of management
1	Manu et al. (2015) [14]	The primary causes of esophageal perforation in adults include iatrogenic procedures, traumatic events, spontaneous ruptures, and the presence of foreign objects.	The array of treatment modalities encompasses nonoperative resuscitation, medical management, endoscopic stenting, thoracoscopic decortication, primary repair supplemented by tissue flap reinforcement, and, in more severe cases, esophageal diversion or resection. Traditional approaches often involve posterolateral thoracotomy followed by primary repair reinforced by additional measures. It is crucial for thoracic surgeons to master the full spectrum of treatment algorithms to ensure optimal patient outcomes.
2	Carrott and Low (2011) [15]	The objective of the surgical intervention is to effectively seal the breach to facilitate esophageal healing and to evacuate esophageal contents from the thoracic, mediastinal, and peritoneal regions.	Available treatments span from nonoperative resuscitation and medical management to endoscopic stenting, thoracoscopic decortication, and primary repair reinforced by a tissue flap, as well as options for esophageal diversion and resection. Conventionally, treatments may involve posterolateral thoracotomy followed by primary repair with additional support. Mastery of these varied treatment algorithms is vital for thoracic surgeons to ensure optimal patient outcomes.
3	Eroglu et al. (2009) [16]	For a long time, a proactive surgical strategy was essential for addressing esophageal perforations.	The choice of surgical method within the first 24 hours with reinforcement by supportive tissue is influenced by the patient's hemodynamic stability, concurrent medical conditions, and the viability of the esophageal tissue for direct repair.
4	Kuppusamy et al. (2011) [17]	In cases where perforation is identified early, there are no indications of sepsis.	Endoscopic treatment has proven to be an effective strategy for managing cases of perforation, particularly when previous treatments have been incomplete.
5	Eroğlu et al. (2018) [18]	A mucosal defect caused by acute esophageal perforation.	Endoscopic clips represent the sole endoluminal tool designed to mend mucosal defects resulting from acute iatrogenic esophageal perforations.
6	Saxena and Khashab (2017) [19]	Closure of iatrogenic perforations	Through-the-scope (TTS) clips, initially developed for hemostasis, are now also employed for the closure of iatrogenic esophageal perforations (perforations that are less than 2 centimeters).
7	Bartell et al. (2020) [20]	Refractory GI hemorrhage, perforations/luminal defects, and fistulas.	The over-the-scope clip (OTSC) system offers a nonsurgical alternative for applying a clip directly to a specific defect. This system uses an applicator cap that is attached to the tip of an endoscope and employs a thread retriever for deployment. For enhanced closure precision, Twin Grasper (TG) or anchor forceps can be utilized to improve the alignment of the tissue edges before clip application. Activation of the clip is achieved by turning the hand wheel on the device, which tightens the thread and deploys the clip onto the defect.
8	Eroğlu et al. (2016) [21]	Benign indications	There has been a noticeable shift from surgical interventions towards an increased reliance on stent placement for treating esophageal perforations.
9	Rogalski et al. (2015) [22]	Esophageal fistula and perforation	Self-expandable plastic stents (SEPS) serve as a minimally invasive solution for managing esophageal fistulas and perforations. Polyflex stents, constructed from polyester and coated with silicone to minimize migration, are the most commonly utilized SEPS. These stents are particularly effective in addressing esophageal leaks and perforations, offering benefits over their metal counterparts.
10	Alzanbagi et al. (2021) [23]	Malignant obstructions	Self-expandable metallic stents (SEMS) are primarily employed in the management of malignant obstructions. The introduction of partially covered fully covered, and removable stents has expanded their applicability.
11	Mastoridis et al. (2022) [24]	Close acute perforations and chronic fistulas	Endoscopic suturing methods are employed for closing larger anomalies such as acute perforations and chronic fistulas. The OverStitch Endoscopic Suturing System, developed by Apollo Endosurgery, provides the capability for both interrupted and continuous suturing and enables the secure attachment of stents to the esophageal wall.
12	Barnett et al. (2023) [25]	Drainage of mediastinal collections	Endoscopic vacuum-assisted closure (EVAC) represents an innovative approach for managing mediastinal collections following an acute esophageal perforation. This technique can be implemented through open surgery, minimally invasive surgical procedures, or interventional radiology practices. Initially designed for the closure of soft tissue defects, the EVAC sponge system has found application in the treatment of perforations. While it was originally developed for managing chronic fistulas, its use has now been extended to include the treatment of acute esophageal perforations.
13	Daneshvar et al. (2017) [26]	Tissue residue and pus	Tissue sealants such as fibrin glue and cyanoacrylate play a crucial role. Fibrin glue is particularly effective in areas devoid of moisture, but its application requires the endoscopic elimination of any tissue remnants and pus. Cyanoacrylate, known for its antibacterial properties, is suitable for use in infected regions. Prior to applying sealants for fistula repair, the mucosal layer around the defect is removed using a cytology brush to promote healing.
14	Sigmon (2024) [27]	Nonoperative management may be used to treat recent iatrogenic or late postemetic esophageal perforations.	Establishing large bore intravenous lines, providing supplemental oxygen, and continuous cardiopulmonary monitoring within a critical care environment without surgery. Patients are advised not to consume any oral intake and should have a nasogastric tube placed for gastric decompression. Initiating broad-spectrum intravenous antibiotics early is critical, with treatment typically lasting 7-10 days. To relieve pain and discomfort, adequate pain management strategies, including the use of narcotics, are essential. Placement of intercostal chest tubes may be required depending on the patient's condition.

Discussions

Esophageal perforation is a complex condition that necessitates the involvement of a multidisciplinary team to provide optimal care. Patients may experience nonspecific symptoms such as abdominal pain, vomiting, sepsis, and shock. These patients should be managed by specialists such as radiologists, intensivists, thoracic surgeons, and endoscopists. Surgical residents should have a high suspicion or diagnosis threshold for esophageal perforations, with radiologists playing an important role in diagnosing complex or small perforations with minimal leaks [8]. This review emphasizes the management of esophageal perforation.

Management strategies for esophageal perforation are categorized into three types: initial, standard, and surgical. The treatment aims to eradicate infection and inflammation, prevent contamination of the mediastinum through drainage and antibiotic therapy, restore the continuity of the alimentary tract, and provide nutritional support. The choice of management approach is influenced by the perforation's mechanism, severity, location, and the time elapsed since the event. Critical factors such as the patient's overall clinical status, the extent of tissue damage, associated injuries, and underlying esophageal conditions must be taken into account. While both nonoperative and operative treatments can be effective, the potential for rapid clinical deterioration necessitates prompt surgical consultation [28,29].

Ben-David et al. (2014) advocated open surgical treatment for esophageal perforations, whereas others have reported success with non-operative treatment and percutaneous control of mediastinal sepsis. As self-expanding covered stents become more common in treating benign perforations and anastomotic leakage, minimally invasive methods are increasingly being used [30,31].

Watkins and Farivar (2018) along with Bufkin et al. (1996) have identified that for stable patients, highlighting the necessity of surgical intervention to diminish morbidity and mortality rates. Early-stage diagnoses may be managed through debridement and primary repair, often enhanced by the addition of a vascularized pedicle flap. Cases presenting with significant fluid leakage, tissue necrosis, or devitalization require urgent surgical action, including stenting, further debridement, or drainage. In exceptional situations, diversionary procedures or resection accompanied by proximal esophagostomy and the establishment of a feeding gastrostomy or jejunostomy might be considered viable options. Additionally, the utilization of a feeding jejunostomy or gastrostomy tube can significantly support the post-surgical recovery process [28,32].

Brinster et al. (2003) and Bhatia et al. (2008) have highlighted that managing esophageal perforation remains a challenging and debated area, emphasizing that early detection and appropriate intervention are crucial for favorable outcomes. Mortality rates can vary between 10% and 50%, largely dependent on the timeliness of the diagnosis. Iatrogenic perforations, often occurring during endoscopic procedures, tend to have more favorable prognoses. The treatment approach is influenced by factors such as the cause and location of the perforation, the patient's overall health, and the extent of radiological contamination. In cases where the diagnosis is made later, nonoperative management may be preferred, although surgery is generally considered the primary treatment strategy. The regimen may include intravenous fluids, broad-spectrum antibiotics, narcotic pain relief, total parenteral nutrition, and surgical intervention over nonoperative approaches. Patients exhibiting hemodynamic instability or compromised airways necessitate care in an intensive care unit (ICU) [33,34].

The location, timing of diagnosis, and the patient's condition all influence esophageal perforation treatment. Cervical perforations are typically treated with surgical drainage or primary repair, resulting in lower morbidity and mortality. Inthoracic perforations require surgical exploration, debridement, buttressed repair, and extensive drainage. Historically, surgical repair has been the norm, especially in early cases. Esophageal diversion may be the best option if primary repair is not feasible due to devitalized tissue. Minimally invasive endoscopic approaches may be most effective for minor perforations, the elderly, or patients with medical conditions [29].

The use of minimally invasive surgery for managing acute esophageal perforations in patients who are stable and have minimal contamination is on the rise. However, the body of literature on this topic is predominantly made up of case reports and small series. Cho et al. (2011) presented a study involving 15 cases of Boerhaave syndrome treated through either thoracoscopy or thoracotomy, discovering that the group undergoing thoracoscopy experienced lower rates of additional operations, ventilator use, and mortality. Therefore, thoracoscopic esophageal repair could be a viable treatment option for stable patients with Boerhaave syndrome or those exhibiting moderate levels of inflammation. Fiscon et al. (2008) implemented a combination of thoracoscopic and endoscopic approaches in treating a case of Boerhaave syndrome. It is standard practice to remove self-expandable plastic stents (SEPS) within 28 days, with stent migration noted as a frequent complication. This issue may be mitigated by opting for stents with larger diameters or by securing the stent's edges to the esophageal wall using endoscopic clips [35,36].

Self-expandable metallic stents (SEMSs) are primarily utilized for managing malignant obstructions, with the advent of partially covered, fully covered, and removable stents broadening their scope of use. Fully covered SEMSs offer protection against contamination from structures outside the esophagus and inhibit tissue re-epithelialization at the perforation site. Partially covered SEMSs, featuring 1.5 cm of uncovered stent at both ends, are designed to reduce the likelihood of migration, though they may require the placement of an additional stent. While fully covered stents are highly effective in preventing leaks, they carry an increased risk of migration. Ensuring proper drainage of the leakage site, especially in cases of perforation, is crucial. However, the use of fully covered stents might obstruct effective drainage of the cavity, potentially leading to sepsis. Freeman et al. (2012) highlighted four key factors that impact the success of stent placement for leakage treatment negatively: leakage occurring in the proximal cervical esophagus, stent extension through the gastro-esophageal junction, esophageal injuries exceeding 6 cm in length, and anastomotic leaks compounded by leaks in more distal parts of the conduit [18,37-39].

In their study of endoscopic vacuum-assisted closure (EVAC), Laukoetter et al. (2017) found a 94% success rate in treating anastomotic leakage and esophageal perforation in 52 cases. Brangewitz et al. (2013) compared 39 patients with SEMP or SEPS to 32 with EVAC for intrathoracic esophageal leakage. They discovered that successful wound closure was independently associated with EVAC treatment, with a higher closure rate (84.4%) in the EVAC group compared to SEMS/SEPS (53.8%). They concluded that EVAC is more effective than stents in closing intrathoracic leaks [39,40].

Minimally invasive techniques offer the advantage of faster patient recovery, alongside reductions in hospital stays, morbidity rates, and healthcare costs. The combination of precise diagnostics and minimally invasive interventions has been shown to decrease both morbidity and mortality rates. For patients in stable condition, with small, adequately drained perforations or chronic esophageal fistulas, endoscopic management is often the treatment of choice. In situations involving significant contamination or extensive, uncontained perforations, a combination of surgical and endoscopic approaches may be employed. As endoscopic and radiological technologies evolve, the integration of hybrid procedures employing various treatment strategies is likely to become more prevalent. Such approaches can be readily adopted by thoracic surgery departments with considerable expertise, potentially leading to a consensus on the best practices for managing esophageal perforations in the foreseeable future [18].

Bhatia et al. (2011) discovered that primary repair is the most common surgical technique and has the lowest mortality rate, even in cases of delayed diagnosis. This is a common trend in larger cities, with no discernible difference in mortality rates between reinforced and unreinforced primary repairs. A recent single-site review discovered an overall operative mortality rate of 8.3%, with primary repair being the most used treatment. The remaining techniques were used infrequently, making the figures more difficult to understand. Although there appears to be a decrease in operative mortality and morbidity, more research is required to determine the extent of this trend [41].

Certain conditions can be successfully managed without surgery, with some patients requiring oral intake cessation, oral hygiene maintenance, antibiotics, nutritional support, and fluid collection drainage. Successful nonoperative management includes cervical tears, intramural dissections, small leaks, and chronic perforation with minimal symptoms. However, up to 20% of nonoperatively treated patients may need surgery, necessitating close monitoring during the first 24 hours of therapy. Nonoperative therapy is safe and effective, with an 18% mortality rate [29].

Endoscopic clipping and stenting are two minimally invasive techniques that have significantly reduced esophageal perforation mortality and morbidity rates. These techniques are now widely used in large centers, up from 0% in 1989 to 75% in 2009. Although the specific indications for these techniques have yet to be formalized, they have proven effective in treating a wide range of aetiologias and esophageal perforation sites in some institutions [17].

Endoscopic clipping and stenting have become prevalent methods for addressing esophageal pathologies and managing small perforations (less than 1.5 cm), particularly when the perforations are clean and show minimal signs of infection. The technique of endoscopically placing coated, self-expanding metal stents has a long history, primarily among high-risk patients or as a palliative approach for obstructive, inoperable tumors. As medical centers accumulate more experience, the criteria for stent placement have expanded to be more inclusive, with some practitioners now viewing stent placement as the primary treatment option in many scenarios. Nonetheless, certain conditions are considered contraindications for stent use, including long segment perforations, perforations identified during thoracotomy or laparotomy, anastomotic leaks accompanied by conduit necrosis or near-complete separation, anastomotic leaks within non-stomach conduits, and cervical esophageal perforations. These contraindications are based on analyses from large medical centers regarding instances where stent placement was unsuccessful and are subject to ongoing review and refinement. Currently, the U.S. Food and Drug Administration (FDA) still classifies the use of esophageal stents for sealing perforations as off-label. There is variability in the type of stent preferred across different practices, with some favoring metal-coated stents, while others opt for silicone-based coverings for the stents [41,42].

## Conclusions

To effectively plan treatment for esophageal perforations, an accurate diagnosis is crucial due to the high morbidity and mortality rates linked to these conditions. Treatment options include non-surgical resuscitation, medical therapy, endoscopic stenting, thoracoscopic decortication, and primary repair with tissue flap reinforcement. In severe cases, esophageal diversion or resection may be necessary. Traditional approaches typically involve posterolateral thoracotomy, primary repair, and additional supportive measures. In order to guarantee the best possible results for their patients, thoracic surgeons must have complete command of these therapeutic algorithms. The patient's hemodynamic stability, history of other medical issues, and the esophagus tissue's viability for direct repair determine the surgical approach. While it is preferred to intervene early on with supporting tissue to help stabilize the patient, this may not be possible for patients who present later in the course of their sickness. Better results have been associated with treatment achieved within the first 24 hours after perforation. There is a lack of study on the effectiveness of through-the-scope (TTS) clips in closing esophageal perforations, despite their widespread use for iatrogenic perforations. One non-invasive option is the over-the-scope clip (OTSC) device, which uses a thread retriever and an applicator cap attached to the endoscope to deploy a clip directly onto a target defect. The most popular SEPS used to treat esophageal perforations and fistulas are Polyflex stents.
